# Epigastric Heteropagus Twin in a Term Neonate: Early Diagnosis, Multidisciplinary Separation, and Favorable Outcome: A Case Report

**DOI:** 10.1055/a-2899-2275

**Published:** 2026-07-06

**Authors:** Abdelrahman S. Elnour, Bushara Abdullah, Leena Abdelmagid, Rammah Bireama, Moataz Idriss, Faisal Nugud

**Affiliations:** 1 Botswana–Baylor Children's Clinical Centre of ExcellenceGaboroneBotswana; 2 Department of Pediatric Surgery 559556 Sudan Medical Specialization BoardKhartoum, KhartoumSudan; 3 Department of Surgery 472715 University of Gezira Faculty of MedicineWad Madani, Al JazirahSudan

**Keywords:** epigastric heteropagus, parasitic twin, neonatal surgery, case report

## Abstract

**Introduction:**

Epigastric heteropagus twinning is an extremely rare form of asymmetric conjoined twinning, characterized by a parasitic twin attached to the upper abdomen of a structurally normal autosite.

**Case Presentation:**

We report a full-term female neonate born via elective cesarean section with an epigastric heteropagus parasitic twin. The parasitic twin had a well-formed head and neck with a rudimentary trunk, and absence of limb structures. The autosite was clinically stable, with normal cardiovascular and respiratory status, and no congenital anomalies were detected on echocardiography. Contrast-enhanced computed tomography revealed a well-formed calvarium and cervical spine in the parasitic twin, absent thoracic and abdominal organs, and two major feeding vessels from the autosite. There was no visceral sharing. Surgical separation was performed on day 10 of life by a multidisciplinary team. Both feeding vessels were carefully ligated, the parasitic twin was completely excised, and the anterior abdominal wall was reconstructed. Postoperative recovery was uneventful; oral feeding resumed on day 3, and the infant was discharged on day 7. At 9-month follow-up, growth and developmental milestones were normal, with no evidence of hernia or other complications.

**Conclusion:**

Early separation of epigastric heteropagus twins is safe and effective when there is no visceral sharing, the vascular anatomy is clearly defined, and multidisciplinary planning is implemented.

## Introduction

Heteropagus, or parasitic twinning, is an exceptionally rare congenital anomaly, with an estimated incidence of fewer than one per million live births and accounting for 1 to 2% of all conjoined twins. 1 2 3 It represents an asymmetric form of conjoined twinning in which a severely underdeveloped parasitic twin is attached to a predominantly normal autosite. Proposed embryologic mechanisms include incomplete fission or secondary fusion of embryonic structures, followed by vascular compromise leading to selective regression of the parasitic twin. 1 2 3 4 5

Epigastric heteropagus is an extremely rare subtype, characterized by attachment of the parasitic twin to the upper abdominal region of the autosite. Early recognition and multidisciplinary planning are essential for safe surgical separation and favorable outcomes. 3 6

We report a case of epigastric heteropagus twinning, describing its antenatal and postnatal evaluation, surgical management, and outcome. This report follows the CARE guidelines ( https://www.care-statement.org ).

## Case Presentation

A 28-year-old woman, gravida 3 para 2, with regular antenatal care, was diagnosed with a twin pregnancy in the first trimester. Antenatal evaluations remained unremarkable until the late third trimester, when detailed ultrasonography revealed a twin with a well-formed head but non-visualization of thoracic, abdominal, and limb structures, raising suspicion for conjoined or heteropagus twinning. Given her prior cesarean section, an elective cesarean delivery at 38 weeks was performed.

A live female infant weighing 3.1 kg was delivered with an epigastric heteropagus parasitic twin. The autosite cried immediately after birth, with Apgar scores of 8 and 9 at 1 and 5 minutes, respectively. The autosite was admitted to the neonatal intensive care unit (NICU). On examination, the infant was clinically stable, with an oxygen saturation of 93% on room air and no respiratory or cardiovascular abnormalities. A parasitic mass was attached to the epigastrium, covered with intact skin, with no ulceration or signs of ischemia. The parasitic twin demonstrated a well-formed head and neck with a rudimentary trunk and complete absence of limb structures, accompanied by active oral movements and salivation ( Fig. 1A, B ).

**Fig. 1:**
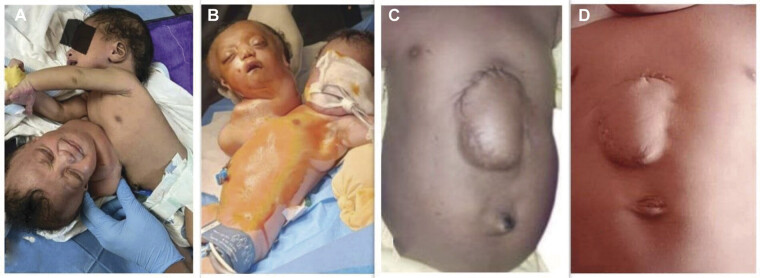
Epigastric heteropagus parasitic twin attached to a structurally normal autosite, showing a well-formed head and neck with a rudimentary trunk and no limbs ( **A, B** ). Postoperative follow-up at 3 months ( **C** ) and 6 months ( **D** ) demonstrates a well-healed anterior abdominal wall without incisional hernia.

Laboratory tests, including complete blood count, liver and renal function tests, were within normal limits. Echocardiography showed normal intracardiac anatomy and ventricular function.

Contrast-enhanced computed tomography (CT) demonstrated a structurally normal autosite. The parasitic twin had a well-formed calvarium and cervical spine, absent thoracic and abdominal organs, and was supplied by two major vessels: one arising from the aortic arch and another from a vessel beneath the heart. No visceral sharing was observed, and the autosite's liver, bowel, diaphragm, and major vessels were normal ( Fig. 2 ).

**Fig. 2:**
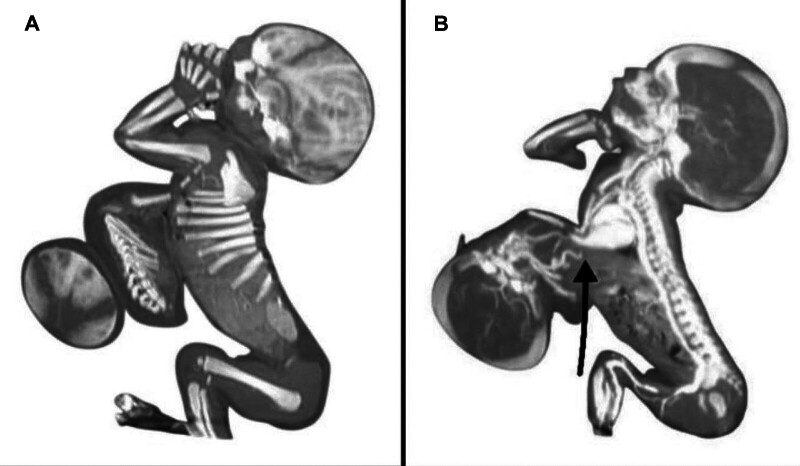
Select views of the contrast-enhanced CT scan showing the parasitic twin's cranial and cervical skeletal structures and its attachment to the autosite ( **A** ). The parasitic twin has a well-formed calvarium and cervical spine and is supplied by two major vessels ( **B** , arrow).

At 10 days of life, surgical separation was performed by a multidisciplinary team comprising pediatric, plastic, and cardiothoracic surgeons, with dedicated pediatric anesthesiology support. Intraoperatively, the parasitic twin was attached by soft tissue, including identifiable vascular pedicles. The feeding vessels were meticulously ligated and divided, allowing complete excision of the parasitic structures. No abdominal muscular or sternal defect was identified at the site of attachment. Reconstruction of the anterior abdominal wall was performed in layers, and skin coverage was achieved using a fasciocutaneous flap. Estimated blood loss was minimal, and no intraoperative complications were encountered.

Postoperatively, oral feeding was initiated on day 3 and was well tolerated. The infant remained clinically stable, with satisfactory wound healing, and was discharged on postoperative day 7. At 9-month follow-up, growth and developmental milestones were appropriate. Examination revealed a residual bulky flap without evidence of hernia or abdominal wall weakness ( Fig. 1C, D ).

## Discussion

Epigastric heteropagus represents one of the rarest forms of asymmetric conjoined twinning, with fewer than 50 cases reported worldwide. 3 Prenatal diagnosis remains challenging, especially in resource-limited settings, but detailed ultrasonography can identify abnormalities. Advanced imaging, including three-dimensional ultrasound, fetal echocardiography, and postnatal CT, is critical to delineate anatomy, assess vascular supply, and guide perinatal planning. 1 7 8 Cesarean delivery is generally recommended due to the size and irregularity of the parasite, reducing the risk of birth trauma. 3

Congenital heart disease is a common anomaly in autosites, reflecting the increased hemodynamic load required to perfuse parasitic tissue. Preoperative cardiac evaluation and optimization are therefore essential. Most literature supports early, but not emergent, surgery to allow thorough assessment, optimal timing, and controlled operative planning. 6 Rarely, urgent intervention may be necessary if there is a high risk of parental abandonment due to social stigma associated with visible abnormalities. 1

Mathur et al reviewed 120 heteropagus twin cases (114 from literature, 6 new), with 9.2% diagnosed antenatally; 90.9% of these were delivered via cesarean section. Associated anomalies included meningomyelocele (31.7%), omphalocele (24.2%), and congenital heart disease (14.2%). Mortality was 10%, with 66.7% of deaths related to cardiac anomalies; postoperative wound complications were rare (3.3%). 1

In the largest review of epigastric heteropagus twins by Zhu et al, omphalocele was present in 63.4% of cases, and congenital heart disease in 26.8%. Connections between twins were mostly limited to soft tissue, cartilage, and vascular pedicles, while true organ sharing was uncommon. Although rare, the literature has reported cases of two parasitic twins attached to a single autosite; however, this was not observed in our case. Surgical separation was technically feasible, with a postoperative survival rate of 92.7%, confirming favorable outcomes. 1 3

Separation of heteropagus twins is a technically demanding procedure, with operative timing guided primarily by the physiological status of the autosite. Urgent intervention is indicated in the presence of cardiorespiratory compromise, whereas delayed separation may permit preoperative optimization in stable patients. Considerable heterogeneity in vascular, soft tissue, and skeletal connections necessitates individualized surgical planning and limits procedural standardization. Favorable outcomes are associated with absent visceral sharing and clearly delineated vascular pedicles, which require meticulous dissection and controlled ligation to mitigate hemodynamic instability and prevent residual parasitic tissue. Despite typically being performed as a single-stage operation, staged approaches may be warranted, and postoperative management often involves additional reconstructive and corrective procedures to address associated anomalies and ensure functional restoration. 2 3

Postoperative complications are infrequent, though wound dehiscence, retained parasitic tissue, incisional hernia, and, rarely, neoplastic transformation in internal remnants have been reported. 1 3 9 A multidisciplinary team approach is essential to achieve safe separation, optimize perioperative management, and ensure favorable long-term outcomes. 8 10

Our patient benefited from the absence of visceral sharing, clearly defined vascular pedicles, and a structurally normal autosite. Early separation at 10 days resulted in an uneventful recovery and normal development, consistent with outcomes reported in the literature.

## Conclusion

Early separation of epigastric heteropagus twins is safe and effective when there is no visceral sharing, the vascular anatomy is clearly defined, and multidisciplinary planning is implemented.

## Notes

Importance for a Pediatric Surgeon Early surgical separation of epigastric heteropagus twins is safe and effective when there is no visceral sharing and the vascular anatomy is clearly defined. This underscores the importance of meticulous preoperative imaging and multidisciplinary planning to achieve optimal surgical outcomes.
